# AURKC Promotes Clear Cell Renal Cell Carcinoma Proliferation Through Upregulation of ERp57

**DOI:** 10.7150/jca.103134

**Published:** 2025-01-13

**Authors:** Yan Liu, Yue Wen, Ziyuan Nie, Li Jia

**Affiliations:** 1 Department of Anesthesiology, The Fourth Hospital of Hebei Medical University, Shijiazhuang, 050000, Hebei, China.; 2 Department of Ultrasound, The Fourth Hospital of Hebei Medical University Hebei, Shijiazhuang, 050000, Hebei, China.; 3 Department of Hematology, The Second Hospital of Hebei Medical University, Shijiazhuang, 050000, Hebei, China.

**Keywords:** aurora kinase C, clear cell renal cell carcinoma, ERp57, proliferation

## Abstract

In recent years, aurora kinase C (AURKC) has emerged as a potential therapeutic target for cancer, having been found to induce proliferation in a variety of cancers. However, at present, its precise mechanism remains unclear. In this study, the specific role of AURKC in renal clear cell carcinoma and its mechanism was investigated. The protein expression levels of AURKC were evaluated in clear cell carcinoma and adjacent normal tissues, followed by prognostic analysis. Subsequently, cell models with knocked-down and overexpressed AURKC were constructed for *in vitro* cell experiments, and tumor-bearing mouse models were constructed to confirm the specific role of AURKC *in vivo*. AURKC was found to be highly expressed in ccRCC, which was associated with poor prognosis. In the *in vitro* experiments, the expression levels of CyclinD1 and proliferating cell nuclear antigen (PCNA) proteins were downregulated after AURKC knockdown, and the cell proliferation ability was found to decrease significantly. After AURKC overexpression, the levels of ERp57 protein expression increased significantly, also significantly enhancing the cell proliferation ability. In addition, AURKC was found to interact with ERp57 and exhibited a colocalization relationship. In the *in vivo* experiments, AURKC downregulation significantly inhibited the expression of ERp57 protein and blocked the growth of tumor tissue in tumor-bearing mice. These results suggest that the abnormal expression of AURKC in ccRCC enhances the expression of ERp57 protein, thereby promoting the proliferation of clear cell renal cell carcinoma. Thus, AURKC shows potential as a target for the treatment of ccRCC.

## 1. Introduction

Renal cell carcinoma is the second most common malignancy in the urinary system, accounting for approximately 2-3% of adult tumors^1^. According to the data, over 350,000 new cases of RCC are diagnosed worldwide each year, with more than 150,000 deaths and the prevalence and detection rate of RCC increasing year by year^2^. ccRCC is the most common subtype of RCC, accounting for approximately 80-90% of RCC cases. Compared with other subtypes, such as chromophobe cell carcinoma and papillary cell carcinoma, ccRCC progresses faster and has a worse prognosis^1^, ^3^. For early ccRCC, partial nephrectomy is the most important treatment method, and the prognosis is good. However, as many as 20-30% of patients have metastatic lesions at the time of initial diagnosis, whose 5-year survival rate is often less than 10%^4^. At present, there is a lack of effective methods for the treatment of metastatic lesions or postoperative tumor recurrence. Existing methods, including conventional chemotherapy, immunotherapy, and targeted therapy, are associated with a range of problems, from a low response rate and significant side effects to a heavy economic burden^5^. Therefore, better understanding the occurrence and development mechanism of ccRCC and exploring new and effective therapeutic targets is urgently needed in the field of RCC research.

AURKC belongs to the serine/threonine protein kinase member of the Aurora family and is a key regulatory factor of the chromosomal passenger complex protein kinase during cell mitosis^6^, ^7^. According to the literature, AURKC is a candidate biomarker for multiple cancers^8^, ^9^. The aberrant expression of AURKC can induce the transformation and oncogenicity of epithelial cells and the growth of ovarian cancer cells^10^, ^11^. In addition, the targeted inhibition of AURKC has been shown to reduce the metastasis of cancer cells^12^, ^13^. Although studies have previously highlighted the potential therapeutic potential of AURKC, the mechanism by which AURKC promotes cancer remains unclear.

Endoplasmic reticulum resident protein 57(ERp57), Identified as protein disulfide isomerase family A, member 3 (abbreviated as PDIA3), it belongs to the protein disulfide isomerase (PDI) gene lineage. It was named after the fact that it was first reported to promote the folding of glycoproteins in the endoplasmic reticulum (ER)^14^. However, the function of ERp57 goes far beyond that in the ER^15^. Research findings have indicated that the expression of ERp57 is frequently disrupted across various forms of malignancies, with either increased or decreased levels of ERp57 linked to unfavorable outcomes in neoplasms^16^-^18^. Notably, ERp57 expression is diminished in the initial stages of cervical cancer, whereas it is elevated in the advanced, invasive stages, suggesting the intricate nature of its function in carcinogenesis and its intimate connection with molecular chaperones^19^, ^20^. In previous studies, we found that ERp57 is upregulated in ccRCC, promoting the transcription of interleukin enhancer binding factor 3 (ILF3) after binding to signal transducer and activator of transcription 3 (STAT3). Furthemore, ILF3 can bind and stabilize the mRNA of ERp57, thereby promoting the expression of ERp57. The feedback loop formed by ERp57/STAT3/ILF3 plays a key role in the proliferation mechanism of ccRCC^21^. However, a correlation between ERp57 and AURKC has yet to be reported. In this study, the interaction between ERp57 and AURKC is described, providing potentially useful insights for use in the treatment of ccRCC.

## 2. Methods and Materials

### 2.1 Cell culture

The cell lines of human ccRCC SKRC-39, Caki-2, ACHN, SW839, and A498 were purchased from the Qingqi (Shanghai) Biotechnology Development Co., Ltd. The cells were removed from liquid nitrogen and resuscitated. Cells were cultured in DMEM (11965092; Thermo Fisher Scientific, China) high sugar medium, comprised of a mixture of 10% fetal bovine serum (FBS) (10100147C; Thermo Fisher Scientific, Australia) and 1% penicillin streptomycin (15140148; Thermo Fisher Scientific, China).

### 2.2 Cellular transfection

Exponentially growing SW839 and SK23-39 cells were used. The old medium was removed, and 1 mL of trypsin (25200114; Thermo Fisher Scientific, China) digestive solution was added and placed in a 37°C, 5% CO_2_ incubator (51023126; Thermo Fisher Scientific, China) for 2 min. The cell suspension was collected in a centrifuge tube and centrifuged at 1200 × *g* for 3 min. Next, 3 mL of DMEM medium was added to resuspend the cell precipitate, and 2 × 10^6^ cells were seeded per well in a six-well plate and incubated overnight. The old medium was removed and replaced with Lipofectamine™ 3000 Transfection Reagent (L3000015; Thermo Fisher Scientific, China) in SW839 and SK23-39 cells, which was then added to the mixed solution of siRNA interference sequence or overexpression vector previously prepared using serum-free DMEM medium. The solutions were then placed in the incubator for 5 h. After incubation, the DMEM medium was changed, and the SW839 cells were divided into the si-NC and si-AURKC groups, while the SK23-39 cells were divided into the oe-NC and oe-AURKC groups for subsequent experiments. The sequences of the siRNA and overexpression vectors were sent to Shanghai Jikai gene medical technology joint stock company (China) for synthesis. The corresponding sequence information is shown in Table S1.

### 2.3 Cell Counting Kit-8 assay

The transfected ccRCC cells were digested and centrifuged as described above. Briefly, 2,000 cells per well were seeded in a 96-well plate and incubated for 0, 12, 24, 48, and 72 h, respectively. After that, the 96-well plate was taken out. Next, 10 μL of CCK8 (CA1210; Solarbio, China) solution was added to each well of the 96-well plate, followed by incubation for 3 h. After incubation, the optical density (OD) at 450 nm (OD_450_) was measured using Multiskan SkyHigh full-wavelength enzyme-labeled instrument (A51119700DPC; Thermo Fisher Scientific, China).

### 2.4 Animals

BALB/c-nu mice (six males, 4-6 weeks, 16-20 g) were purchased from Henan SKBES Biotechnology Co., Ltd (China). The rodents were kept in a sanitized atmosphere, experiencing a 12-hour lighting/daytime rhythm, and had unlimited access to germ-free water and nourishment. These animals were nurtured within the confines of the Animal Research Facility at Hebei Medical University's Fourth Hospital (SYXK2022-011), and were adaptively fed for one week after purchase before starting the relevant experiments. Exponentially growing SW839 cells were subcutaneously injected into the backs of the mice at a dose of 1 × 10^7^ per mouse. On the third day after injection, the mice were randomly divided into a control group and a treatment group, with three mice in each group. The experimental cohort received Danusertib via intraperitoneal administration at a dosage of 15 milligrams per kilogram per day, on an alternating three-day schedule, whereas the comparator cohort was administered an equivalent amount of physiological saline solution containing hydrochloric acid^22^. Every three days, the body weight, length, width, and height of the tumors were measured and recorded. On day 15 day after inoculation, the mice were euthanized by cervical dislocation, and the tumor tissues of the mice were removed for subsequent experimental studies.

### 2.5 Hematoxylin and eosin (HE) staining

In this study, we used the HE staining method to detect 40 cases of clear cell renal cell carcinoma patients and adjacent tissues. According to the instructions of the HE staining kit (C0105S; Beyotime, China), the tumor tissue was placed in the fixing solution (P0098; Beyotime, China) and incubated at room temperature for 48 h. After dehydration, the tissue samples were embedded in paraffin. The corresponding paraffin sections (4 μm) were placed in xylene for dewaxing, followed by rehydration in a gradient of ethanol. Then, hematoxylin was added for staining for 5 min, and eosin was added for staining for 1 min. The section was dehydrated and sealed.

### 2.6 Clinical sample collection

All specimens of ccRCC were sourced from Hebei Medical University's Fourth Hospital. The patients' clinical and pathological details were gathered from the hospital's medical records database, with prior informed consent secured from all participants for the use of human samples. No patient had undergone neoadjuvant chemotherapy, radiation therapy, or any form of preoperative targeted treatment. The ethical review board of the hospital granted authorization for the utilization of these samples and associated data (ethical approval code: 2020KY188).

### 2.7 Immunohistochemistry

In this study, we employed the immunohistochemical method to examine 40 cases of clear cell renal cell carcinoma patients and adjacent tissues. Tissue samples were fixed, embedded, and sliced as previously described. Antigen repair solution (P0081; Beyotime, China) was added to the tissue slices and incubated in a water bath at 98°C for 15 min.Following the process of spontaneous cooling, a goat serum sealing reagent (product code C0265; supplied by Beyotime, based in China) was introduced and allowed to react for a duration of 10 min at ambient temperature. Subsequently, the primary antibody AURKC (catalog number D261347, diluted at a ratio of 1:100; produced by Sangon Biotech, a Chinese company) was applied and left to interact with the sample for an entire night at a temperature of 4 degrees Celsius. Post this, the tissue sections underwent a triple rinse with PBS before being exposed to the relevant secondary antibody (identified by the code S0001, diluted at a ratio of 1:100; provided by Affinity Biosciences, headquartered in China) for a period of 30 min. DAB solution (P0202; Beyotime, China) was then added and incubated for 3 min, followed by the addition of hematoxylin (C0107; Beyotime, China) and incubation for 2 min. After dehydration, a sealing solution (C0181; Beyotime, China) was added for sealing, and the slice was allowed to air dry naturally.

### 2.8 Immunofluorescence

The tissue was fixed, embedded, and sliced as previously described. The permeabilization agent for tissue cells was introduced and left to sit at ambient temperature for a duration of 20 min. Subsequently, a 10% concentration of goat serum was incorporated and allowed to interact for a full hour. The initial antibody (catalog number 38-9400, diluted 1:100; supplied by Thermo Fisher Scientific, based in China) was subsequently applied and subjected to an overnight incubation period. Following this, the conjugate antibody (identified as A-11037, diluted 1:100; also from Thermo Fisher Scientific, China) was introduced and kept at room temperature for a period of 60 min. The sections were then subjected to a triple rinse process using PBS, and DAPI (62248; Thermo Fisher Scientific, China) was dropped and incubated in the dark for 15 min. After sealing, the slices were allowed to air dry naturally.

### 2.9 RT-qPCR (Reverse Transcription- quantitative PCR)

Tissue and cellular total RNA was isolated utilizing the TriZol reagent (product code 15596026CN; Thermo Fisher Scientific, China) as per the provider's guidelines. Subsequent to quantifying the RNA, the SuperScript™ IV First Strand Synthesis Kit (Thermo Fisher Scientific, catalog number 18091050, China) was employed as directed. The synthesis reaction was conducted at a temperature of 50°C for a duration of 10 min to generate cDNA. The FastKing One-Step RT-PCR Kit (code KR123; Tiangen Biochemical Technology Co., Ltd., China) was then utilized, incorporating specific primers into the cDNA template. This involved the addition of 2× FastKing One-Step RT-PCR MasterMix, 25 × RT-PCR Enzyme Mix, and ddH20 to reach a final volume of 50 μL. The PCR amplification protocol comprised of an initial denaturation at 42°C for 30 min, followed by a further denaturation at 95°C for 3 min, then 39 cycles of amplification including denaturation at 94°C for 30 seconds, annealing at 60°C for 30 seconds, and extension at 72°C for 30 seconds, culminating with a final extension at 72°C for 5 min. The RT-qPCR primer sequences are provided in Table S1.

### 2.10 Western blotting

Lysis buffer (P0013B; Beyotime, China) was added to the tumor tissues and cells before incubating on ice for 30 min. Then, the protein solution was placed in a 98°C water bath for 15 min. The protein concentration was determined using a BCA protein quantification kit (P0009; Beyotime, China), and the lysis buffer and loading buffer (P00015L; Beyotime, China) were supplemented according to the protein concentration. Gel electrophoresis was performed, and an equal amount of protein solution was added to the loading well. The electrical potential was adjusted to 80 volts for a duration of half an hour, followed by an increase to 120 volts for one hour. Post-electrophoretic procedures involved a transfer process, during which the electrical current was maintained at 260 milliamps for a period of 90 min. Subsequently, the PVDF membrane was submerged in a 5% solution of non-fat milk powder (catalog number P0216; supplied by Beyotime, a company based in China) and subjected to incubation for two hours. Next, primary antibody was added and the membrane was incubated at 4°C overnight. Then, the secondary antibody was added, followed by incubation for 2 h. Finally, the membrane was washed in Tris Buffered Saline with Tween-20 three times. Next, chemiluminescent fluid was dropped and placed in a gel imager for photography, and the photos were saved for Image J 1.52a statistical analysis. The following antibodies were used: AURKC (38-9400, 1:1000; Thermo Fisher Scientific, China), CyclinD1 (ab134175, 1:1000; Abcam, China), PCNA (ab29, 1:1000; Abcam, China), ERp57 (ab13506, 1:1000; Abcam, China), β-actin (ab8226, 1:2000; Abcam, China), GADPH (ab8245, 1:2000; Abcam, China), goat anti-rabbit (A0277, 1:5000; Beyotime, China), goat anti-mouse (A0286, 1:5000; Beyotime, China).

### 2.11 Co-immunoprecipitation (COIP)

Cell lysis was performed as previously described. Briefly, the primary antibody was added to 200 μL of cell lysate and rotated overnight at 4°C to form a protein complex, according to the manufacturer's requirements (P2175M; Beyotime, China). The lysate and antibody solution were transferred to a tube containing magnetic bead sediment, and rotated and incubated at room temperature for 20 min. Then, the magnetic beads were separated using a magnetic separation stand. The sediment was washed with cell lysate 5 times, after which 40 μL of protein loading buffer was added and mixed by rotation. Subsequently, the gel electrophoresis operation was performed as previously described.

### 2.12 Statistical analysis

Data were analyzed using GraphPad Prism 9.5.0. The statistical significance of AURKC expression in paired samples was evaluated using a paired-samples t-test. Using the log-rank test, the survival curves of the two groups were compared, and the resulting test statistic approximately followed the X^2^ distribution with the degree of freedom of the number of groups (1 in large samples). Unpaired t-tests were used to compare data between the two groups. Variability among several clusters was assessed via a univariate analysis of variance (ANOVA) followed by Tukey's post-experimental analysis. To evaluate the data across diverse intervals, Bonferroni's post-experimental adjustment was applied. A p-value below 0.05 was deemed to indicate statistical significance.

## 3. Results

### 3.1 AURKC is upregulated in ccRCC tissues and correlates with poor prognosis

**Figure 1:**
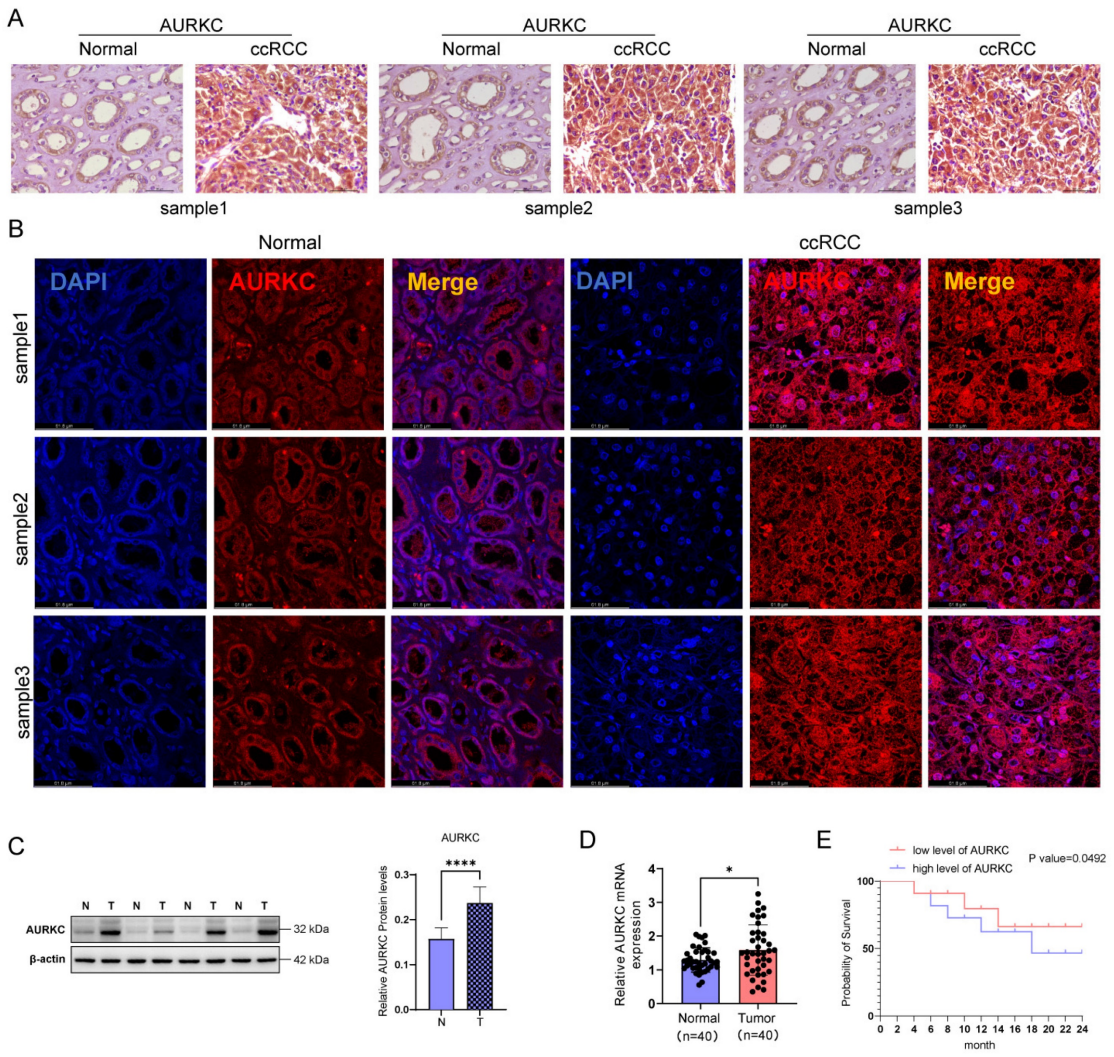
** AURKC is aberrantly expressed in ccRCC cells.** (A) Immunohistochemical analysis of AURKC protein levels in ccRCC and adjacent tissues. Scale bar = 50 μm. (B) Immunofluorescent staining was used to locate and detect the expression of AURKC in A498 cells. Scale bar = 61.8 μm. (C) AURKC protein levels in ccRCC and adjacent tissues was detected by western blotting. (D) RT-qPCR of AURKC protein levels in ccRCC and adjacent tissues. (E) Prognosis survival analysis of AURKC. *P < 0.05, ****P < 0.0001 vs. adjacent tissues.

To evaluate the role of AURKC in ccRCC, we first detected the expression of AURKC in ccRCC (n=40) and adjacent tissues (n=40) using immunohistochemistry and immunofluorescence staining. As shown in Figure 1A, compared with the adjacent tissues, the protein level of AURKC was significantly increased in ccRCC tissues. Immunofluorescence staining also confirmed that the expression of AURKC was significantly increased in ccRCC tissues (Figure 1B). In addition, the western blotting results also showed that the protein expression of AURKC in ccRCC tissues was significantly higher than that in adjacent tissues (Figure 1C). The RT-qPCR results showed that the mRNA levels of AURKC in ccRCC tissues was significantly higher (approximately 3 times) than that in the adjacent tissues (Figure 1D). These results indicate that AURKC is upregulated in ccRCC tissues. Further, according to the expression levels of AURKC mRNA, 40 patients with ccRCC were divided into two groups (a high-expression group and a low-expression group). Then, survival analysis was performed on the two groups. The results shown in Figure 1E indicate that patients with high AURKC expression had a significantly worse prognosis than those with low levels of expression. These findings confirm that the expression of AURKC was increased in patients with ccRCC, suggesting that high levels of AURKC expression are closely related to poor prognosis.

### 3.2 AURKC is involved in ccRCC cell proliferation

**Figure 2:**
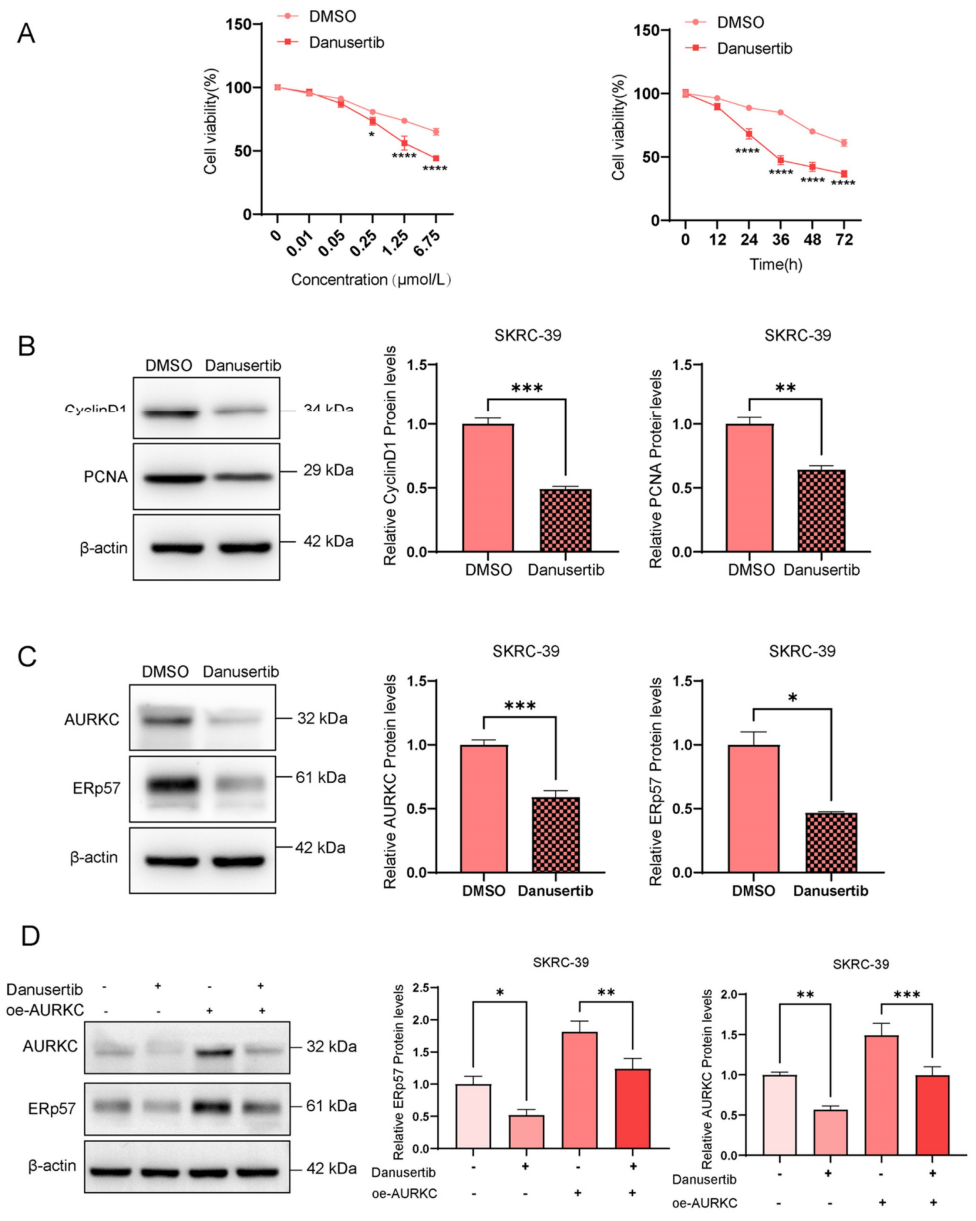
** AURKC promotes the proliferation of ccRCC cells.** (A) RT-qPCR was used to detect the AURKC mRNA levels in five ccRCC cell lines (SKRC-39, Caki-2, ACHN, SW839, and A498). (B) In SW839 cells, after transfection with siAURKC, the expression levels of AURKC protein and mRNA were detected by western blotting and RT-qPCR. (B)(D) Utilizing western blot analysis and RT-qPCR, the quantities of AURKC protein and its mRNA were assessed in SKRC-39 cells that had been transfected with oeAURKC. (F) employing CCK8 methodology, the cellular proliferation rate was evaluated. (G) In SW839 cells treated with siAURKC and SKRC-39 cells treated with oeAURKC, the protein concentrations of PCNA and Cyclin D1 were analyzed through western blotting. ***Indicates P < 0.001, ****Indicates P < 0.0001, when compared to the respective control groups.

To further investigate the role of AURKC in the proliferation of ccRCC cells, we conducted relevant biological function experiments. To this end, RT-qPCR was used to detect the AURKC mRNA expression levels in five different ccRCC cell lines. As shown in Figure 2A, the results demonstrated that among the five ccRCC cell lines, the AURKC mRNA expression level was the lowest in the SKRC-39 cell line, while the ERp57 mRNA expression level in the SW839 cell line was significantly higher than that in the other cells. Consequently, in the subsequent *in vitro* cell function experiments, the SKRC-39 cell line was selected for AURKC overexpression, while the SW839 cell line was selected for AURKC knockdown. Western blot analysis revealed that compared with the control group, the transfection of si-AURKC in SW839 cells significantly downregulated the protein and mRNA expression levels of AURKC in the cells (Figure 2B). In contrast, we synthesized the AURKC overexpression plasmid pWPI-AURKC and transfected it into the SKRC-39 cells. As shown in Figure 2C, after the transfection of pWPI-AURKC in SKRC-39 cells, the expression levels of AURKC protein and mRNA in the cells were significantly increased with statistically significant results. Furthermore, we employed the CCK-8 assay to evaluate the role of AURKC in cell proliferation. As shown in Figure 2D, compared with the control group, the knockdown of AURKC expression in the SW839 cells significantly inhibited cell proliferation, while the overexpression of AURKC in the SKRC-39 cells promoted cell proliferation with statistically significant results. Additionally, the results of western blotting analysis demonstrated that compared with the control group, the knockdown of AURKC in the SW839 cells significantly downregulated the levels of the signature proteins of cell proliferation, namely Cyclin D1 and PCNA. By contrast, the overexpression of AURKC in the SKRC-39 cells promoted cell proliferation. These findings suggest that AURKC functions as an oncogene and is involved in the proliferation of ccRCC (Figure 2E).

### 3.3 AURKC interacts with ERp57 in ccRCC cells

**Figure 3:**
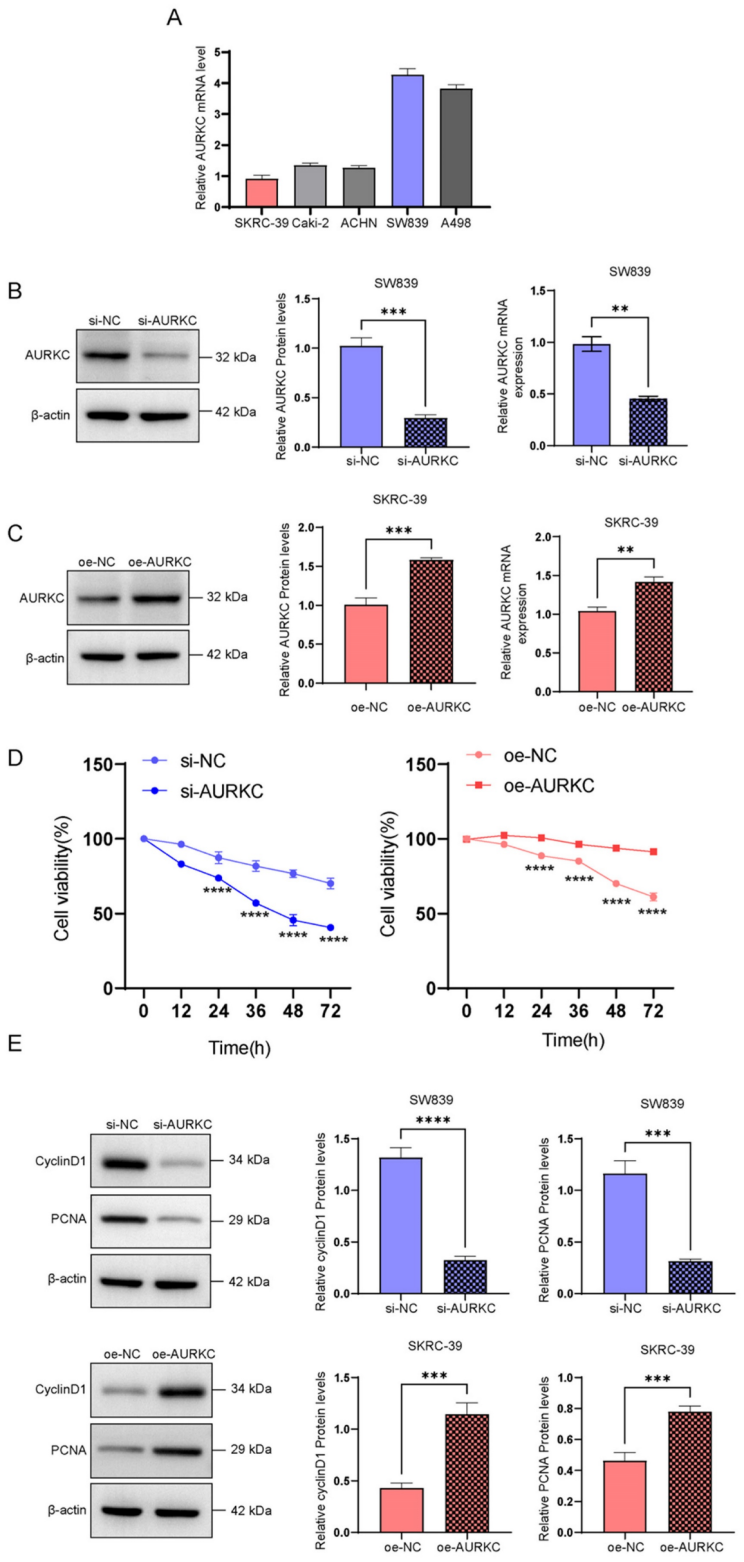
** AURKC promotes ccRCC cell proliferation.** (A-B) In SW839 and SKRC-39 cells, the potential interaction between AURKC and ERp57 was verified via COIP assays. Utilizing immunofluorescence labeling techniques, the distribution and quantitative expression of ERp57 and AURKC proteins were identified within SW839 cellular cultures and clear cell renal cell carcinoma (ccRCC) tissue samples. Measurement scale: 61.8 micrometers.

To clarify the interaction between AURKC and ERp57 in ccRCC cells and confirm their relationship, we performed a co-immunoprecipitation (COIP) experiment. As shown in Figure 3A and B, in SW839 and SKRC-39 cells, co-precipitation was performed using an AURKC antibody. ERp57 protein was detected in the resulting precipitate, indicating that ERp57 directly binds to AURKC to form a complex. Then, immunofluorescence double staining was used to confirm the interaction between the two. As shown in Figure 3C and D, the co-localization of AURKC and ERp57 was observed in the cytoplasm of ccRCC cells and tissues. These results indicate that in ccRCC cells, AURKC and ERp57 interact to form a complex.

### 3.4 AURKC enhances the expression level of ERp57 protein

**Figure 4:**
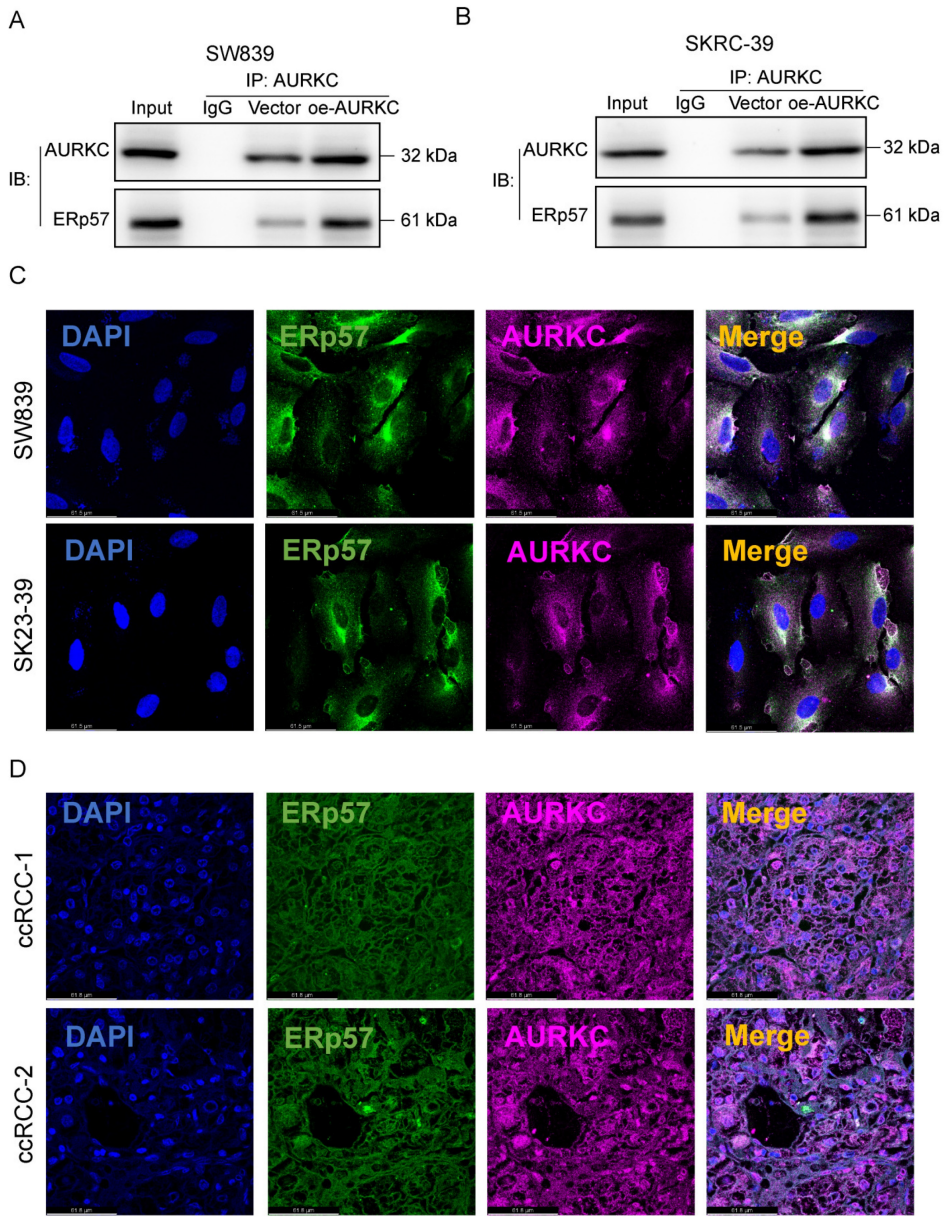
** AURKC enhances the expression level of ERp57.** (A) ERp57 mRNA levels were detected by RT-qPCR in SW839 cells transfected with siAURKC and SKRC-39 cells transfected with oeAURKC. (B) The expression level of ERp57 protein in SW839 transfected with siAURKC was detected by western blotting. (C) The expression level of ERp57 protein in SKRC-39 cells transfected with oeAURKC was detected by western blotting. (D) The protein expression levels of ERp57 in the cytoplasm and nucleus of SW839 cells transfected with siAURKC were detected by western blotting. ***P < 0.001, ****P < 0.0001, vs. corresponding control.

To further confirm the regulatory effect of AURKC on ERp57, the RT-qPCR results indicated that AURKC knockdown significantly reduced the expression levels of ERp57 mRNA, while AURKC overexpression upregulated the ERp57 mRNA expression levels (Figure 4A). Subsequently, we knocked down the expression of AURKC in SW839 cells and detected the protein expression level of ERp57 using western blotting. The results showed that knocking down AURKC in SW839 cells significantly downregulated the expression levels of ERp57 protein; conversely, overexpressing AURKC in SKRC-39 cells significantly upregulated the ERp57 protein expression levels (Figure 4B and C). We also found that the protein expression level of ERp57 in the nucleus and cytoplasm was significantly reduced (Figure 4D). Taken together, these results indicate that knocking down AURKC inhibits the level of ERp57 in the cytoplasm and nucleus of ccRCC cells.

### 3.5 Danusertib exerts anti-tumor effects by inhibiting the AURKC/ERp 57 pathway

**Figure 5:**
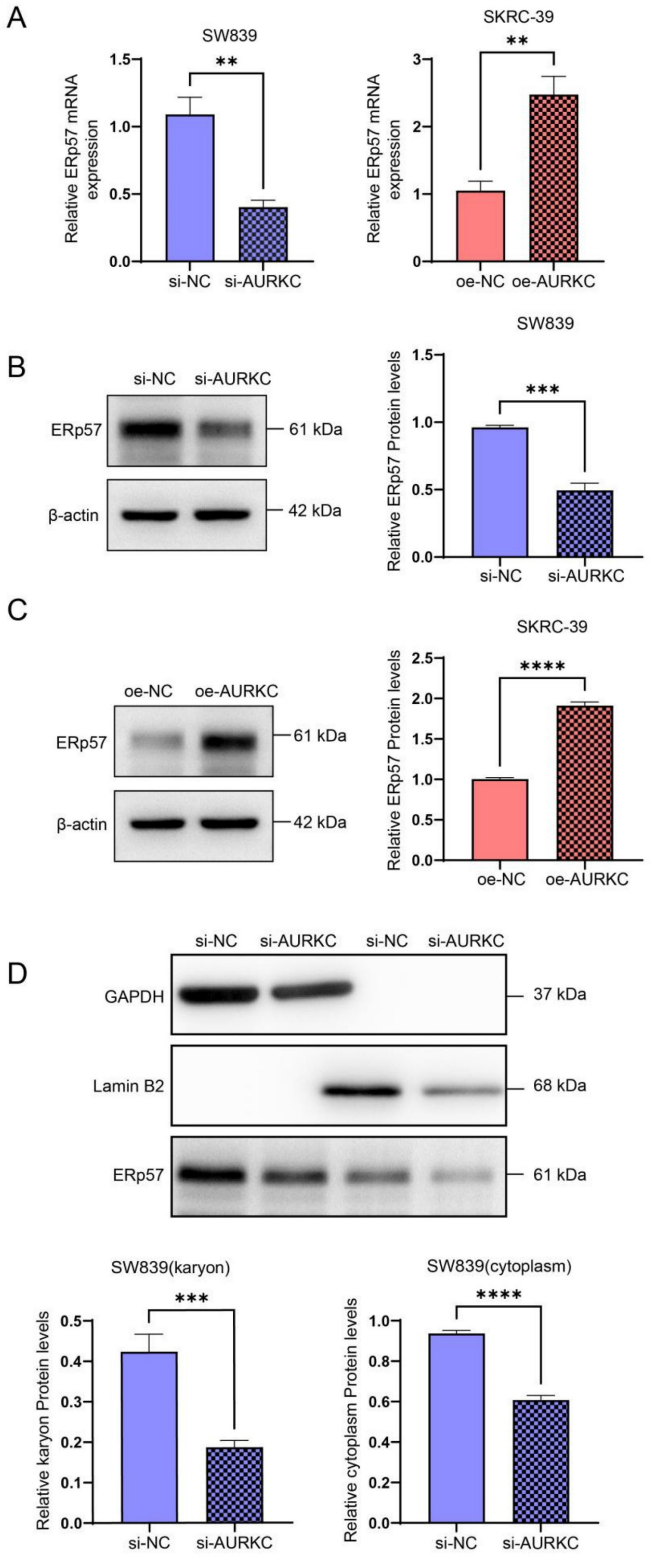
** Danusertib inhibits the AURKC/ERp57 pathway and ccRCC cell proliferation.** (A) Cell viability was detected via CCK8 assays after treating SKRC-39 cells with Danusertib at different concentrations (0.01, 0.05, 0.25, 1.25, 6.75 μmol/L) or time points (12, 24, 36, 48, 72 h). (B-C) After treating SKRC-39 cells with Danusertib for 48 h, the relative expression levels of CyclinD1, PCNA, AURKC, and ERp57 proteins were detected by western blotting. (D) After pre-treating SKRC-39 cells with Danusertib for 48 h, the oeAURKC overexpression vector was introduced into the cells and incubated for a period of 48 hours. Subsequently, the protein expression levels of ERp57 and AURKC were assessed using western blot analysis. Statistical significance is indicated as follows: *P < 0.05, **P < 0.01, ***P < 0.001, vs. corresponding control.

Previous studies have confirmed that the AURKC/ERp57 pathway plays an important role in the proliferation of ccRCC. In the present study, we sought to confirm whether the small molecule inhibitor of AURKC can exert anti-tumor effects by inhibiting the AURKC/ERp57 pathway. Danusertib, an aurora kinase inhibitor, has been shown to exert anti-tumor effects in several types of tumor cells^23^. To evaluate this, we added different concentrations of Danusertib (0, 0.01, 0.05, 0.25, 1.25, and 6.25 μmol/L) to SW398 cells and stimulated them for 24 h before detecting cell proliferation using CCK8 assays. The results showed that Danusertib significantly inhibited the proliferation of ccRCC cells compared with the negative control group, with a half-maximal inhibitory concentration (IC50) of 2.99±0.4818 μmol/L (Figure 5A). Furthermore, to evaluate cell proliferation at different time points using CCK-8, 2.99 μmol/L of Danusertib was added to SW398 cells. The results showed that Danusertib significantly inhibited the proliferation of ccRCC cells in a time-dependent manner (Figure 5B). In addition, after adding Danusertib to SW839 cells for 48 h, the results of western blotting analysis showed that Danusertib significantly downregulated the protein expression levels of cell proliferation-related proteins PCNA and Cyclin D1 compared with the control group. These results indicate that Danusertib is capable of inhibiting the proliferation of ccRCC cells (Figure 5B). To further verify that Danusertib exerts anti-tumor effects by inhibiting the AURKC/ERp57 pathway, we added Danusertib to SW839 cells and detected the expression of ERp57 by western blotting. As shown in Figure 5C, the addition of Danusertib significantly downregulated the expression of ERp57 in cells; in addition, the remedial experiment also confirmed that the addition of Danusertib significantly inhibited the expression of ERp57. However, when the cells were overexpressed with AURKC, this inhibitory effect was weakened. These results suggest that Danusertib inhibits the proliferation of ccRCC cells by suppressing the expression levels of AURKC and ERp57 proteins (Figure 5D).

### 3.6 Inhibition of ccRCC tumor growth by Danusertib in *in vivo* experiments

**Figure 6:**
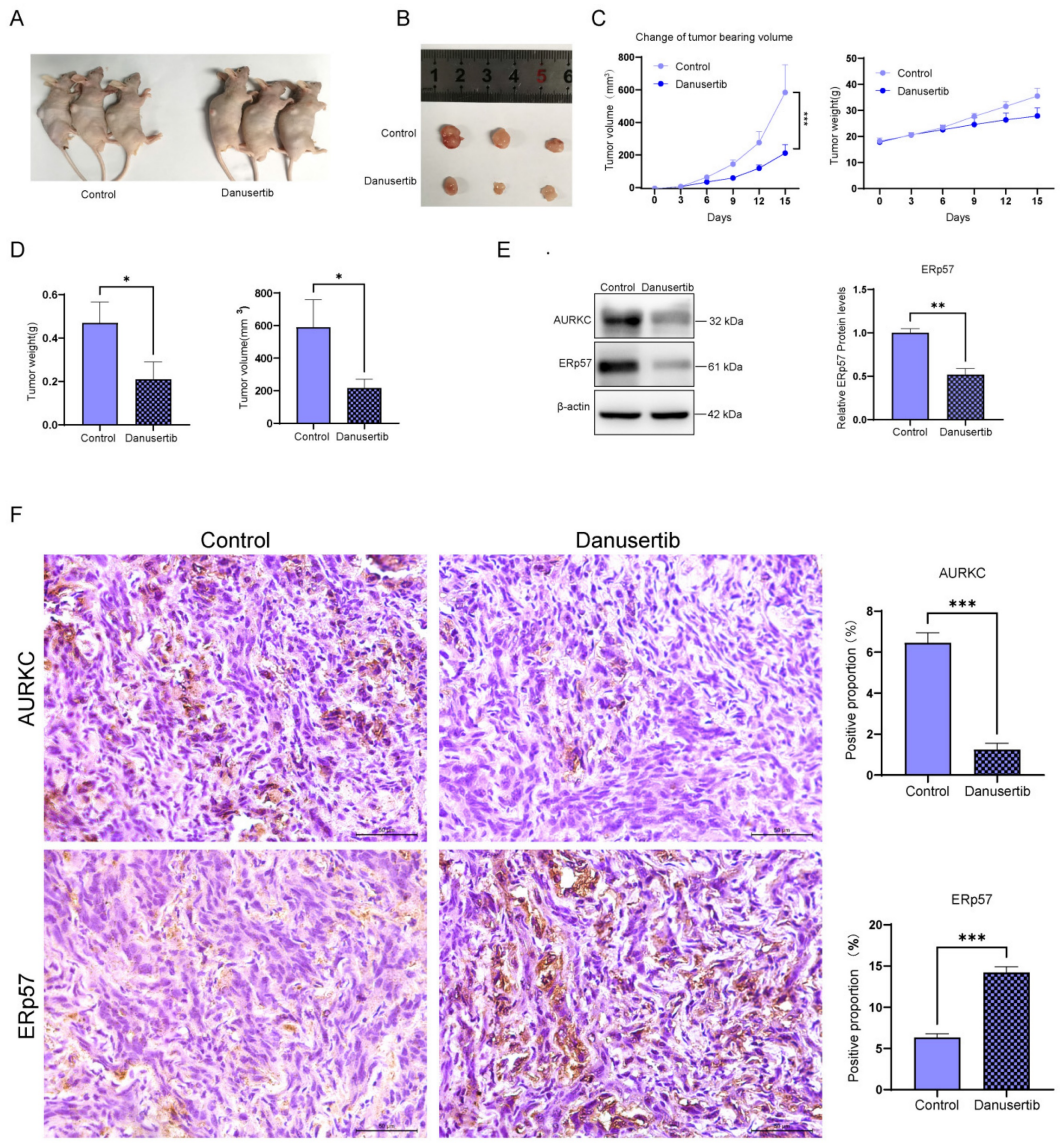
** Danusertib inhibits the AURKC/ERp57 pathway and ccRCC cell proliferation.** (A-B) SW839 cells were injected subcutaneously into nude mice to establish ccRCC xenograft tumors, which was followed by treatment with Danusertib or normal saline. Images were captured to obtain a top-down view of the tumor-bearing mice and the subcutaneous tumor tissue after autopsy. (C) Line graph of tumor tissue weight and volume over time during Danusertib treatment, measured every three days. (D) After dissecting the tumor tissue of the tumor-bearing mice, the tumor volume and tumor size were measured. (E) The relative expression levels of AURKC and ERp57 proteins in the tumor tissue were detected by western blotting. (F) Immunohistochemistry of AURKC and ERp57 protein levels in xenograft tumor. Scale bar = 50 μm. *P < 0.05, **P < 0.01, ***P < 0.001, ****P < 0.0001, vs. corresponding control.

Although the results indicated that Danusertib acts as an AURKC inhibitor, to further confirm the role of Danusertib in ccRCC, we conducted an *in vivo* tumorigenicity experiment in nude mice. After the tumor-bearing mice were sacrificed, a top-down photograph was taken (Figure 6A) showing the subcutaneous tumor tissue (Figure 6B). After injecting the nude mice with SW893 cells, the tumor volume and weight were found to be significantly reduced after the administration of Danusertib compared with the normal saline control group (Figure 6C and D). Next, the levels of AURKC and ERp57 protein expression were detected by western blotting (Figure 6E) and immunohistochemistry (Figure 6F), the results of which indicate that the expression of AURKC and ERp57 in the Danusertib group was significantly decreased. These findings indicate that Danusertib is capable of inhibiting the proliferation of ccRCC in *in vivo* experiments.

## 4. Discussion

The identification of cancer prognostic factors is a key approach in the development of highly efficient targeted drugs^24^. In the present study, high levels of AURKC expression were found to be associated with poor prognosis in ccRCC. AURKC was found to be abnormally expressed in both ccRCC tissues and cells, which is consistent with the results of previous studies on AURKC. Herein, we successfully demonstrated that downregulating AURKC can inhibit the proliferation of ccRCC cells and tissues using *in vitro* and *in vivo* experiments. CyclinD1 is known to be a key regulator of the progression of the G1 phase of the cell cycle, binding to CDK4 or CDK6 to form a complex that activates the cell cycle^25^. PCNA is widely expressed in the S phase and binds to DNA polymerase to participate in DNA replication^26^. Abnormally expressed CyclinD1 is known to be closely related to the occurrence and development of various cancers^27^-^29^. In fact, CyclinD1 overexpression may lead to uncontrolled cell proliferation, thereby promoting the formation of cancer^30^, ^31^. The expression levels of PCNA are also often used as an indicator of cell proliferation activity, and plays a significant role in cancer diagnosis and prognosis^32^. Our findings indicate that the downregulation of AURKC can significantly inhibit the protein expression levels of CyclinD1 and PCNA, thereby inhibiting the proliferation of ccRCC cells.

To further elucidate the regulatory mechanism of AURKC, we turned our attention to ERp57. Numerous studies have indicated that ERp57 is mainly distributed in the cytoplasm, with a small amount entering the nucleus^15^. However, in the presents study, an increase in the nuclear distribution of ERp57 was observed in tumor cells. Based on this finding, we investigated the mechanism underpinning the regulation of the nuclear entry of ERp57. Changes in the intracellular distribution of proteins are often due to modifications, such as phosphorylation, alterations in their sequences, or interactions with other molecules^33^. Hence, we wondered whether ERp57 may be driven into the nucleus by interactions with other protein molecules. Previously, some studies have found that ERp57 can interact with multiple protein molecules; among these, one specific protein, aurora kinase C (AURKC), caught our attention^18^. In subsequent immunoprecipitation experiments, we confirmed that AURKC interacts with ERp57 (Figure 3A and B). In fact, we found that after knocking down AURKC in ccRCC or following treatment with a phosphokinase inhibitor, the expression level of ERp57 was significantly downregulated, and the distribution of ERp57 in the nucleus also decreased, providing evidence in support of our hypothesis.

The main result of this study is the discovery that AURKC interacts with ERp57, with the overexpression of AURKC enhancing the levels of ERp57 protein expression to induce the abnormal proliferation of ccRCC. Although these results are exciting, it is worth highlighting the potential limitations of this study. Whether AURKC, as a serine/threonine protein kinase, promotes the entry of ERp57 into the nucleus by phosphorylating and modifying it after interacting with it, the mechanism underlying this process remains unclear and requires further experimental verification. Furthemore, whether inhibiting AURKC to induce ERp57 can inhibit the invasion and metastasis of ccRCC cells is also unclear, and additional experiments, such as invasion and metastasis assays, will be needed to clarify this. In our previous study, we found that ERp57 was upregulated in ccRCC and promoted the transcription of ILF3 after binding to STAT3. At present, whether AURKC is involved in this mechanism is not clear; however, we plan to make this the focus of future research.

## Conclusion

In this study, the existence of a correlation between AURKC and ERp57, as well as their ability to promote the progression of ccRCC, was demonstrated. Taken together, these results indicate that AURKC may serve as a prognostic biomarker for ccRCC, providing a theoretical basis for the development of new anti-cancer targeted drugs.

## Supplementary Material

Supplementary table.
