# Supplementary material for: AURKC Promotes Clear Cell Renal Cell Carcinoma Proliferation Through Upregulation of ERp57
Source: J Cancer. 2025 Jan 13;16(4):1215–27. doi: 10.7150/jca.103134 (PMC11786039; doi:10.7150/jca.103134)
Supplement: Supplementary file 1 — Supplementary table. [file jcav16p1215s1.pdf]

Table S1 QPCR Primer sequences and the siRNA interference sequences and the AURKC overexpression sequences

Gene	Forward Primer (5′–3′)	Reverse Primer (5′–3′)
AURKC	CCTGGAGGAGGAGGTCCG	AGCTTGCTCTTGGCCATCTT
GAPDH	GAGACCTTCAACACCCCAGC	GATAGCACAGCCTGGATAGCA
Gene	SS Sequence	AS Sequence
siNC	UUCUCCGAACGUGUCACGU	ACGUGACAGGUUCGGAGAA
siRAURKC-1	CCUUGUUCUGCACCCUCAAGC	UUGAGGGUGCAGAACAAGGUG
siAURKC-2	GCUGCGUUCACCUACCCAACU	UUCUCUUCUUGUAUUUCCCAA
siAURKC-3	AGUUCUGAAUGUUAAACUACU	UUGGGUAGGUGAACGCAGCAU
Gene	Primer sequence (5 '-3')	
pcDNA3.1-R AURKC-F	GGTACCGAGCTCGGATCCATGAGCAACCTGAAGCC	
pcDNA3.1-R AURKC-R	TGGATATCTGCAGAATTCCTAACAGAACTGACGG	
